# Effects of Foam Microbubbles on Electrical Resistivity and Capillary Pressure of Partially Saturated Porous Media

**DOI:** 10.3390/molecules25153385

**Published:** 2020-07-26

**Authors:** Abdulrauf R. Adebayo, Abubakar Isah, Mohamed Mahmoud, Dhafer Al-Shehri

**Affiliations:** 1 Center for Integrative Petroleum Research, King Fahd University of Petroleum & Minerals, Dhahran 31261, Saudi Arabia; 2 Petroleum Engineering Dept., King Fahd University of Petroleum & Minerals, Dhahran 31261, Saudi Arabia; abuibnisah@yahoo.com (A.I.); mmahmoud@kfupm.edu.sa (M.M.); alshehrida@kfupm.edu.sa (D.A.-S.)

**Keywords:** archie equation, foam bubbles, foam texture, critical water saturation, resistivity index, porous media

## Abstract

Laboratory measurements of capillary pressure (*P_c_*) and the electrical resistivity index (*RI*) of reservoir rocks are used to calibrate well logging tools and to determine reservoir fluid distribution. Significant studies on the methods and factors affecting these measurements in rocks containing oil, gas, and water are adequately reported in the literature. However, with the advent of chemical enhanced oil recovery (EOR) methods, surfactants are mixed with injection fluids to generate foam to enhance the gas injection process. Foam is a complex and non-Newtonian fluid whose behavior in porous media is different from conventional reservoir fluids. As a result, the effect of foam on *P_c_* and the reliability of using known rock models such as the Archie equation to fit experimental resistivity data in rocks containing foam are yet to be ascertained. In this study, we investigated the effect of foam on the behavior of both *P_c_* and *RI* curves in sandstone and carbonate rocks using both porous plate and two-pole resistivity methods at ambient temperature. Our results consistently showed that for a given water saturation (*S_w_*), the *RI* of a rock increases in the presence of foam than without foam. We found that, below a critical *S_w_*, the resistivity of a rock containing foam continues to rise rapidly. We argue, based on knowledge of foam behavior in porous media, that this critical *S_w_* represents the regime where the foam texture begins to become finer, and it is dependent on the properties of the rock and the foam. Nonetheless, the Archie model fits the experimental data of the rocks but with resulting saturation exponents that are higher than conventional gas–water rock systems. The degree of variation in the saturation exponents between the two fluid systems also depends on the rock and fluid properties. A theory is presented to explain this phenomenon. We also found that foam affects the saturation exponent in a similar way as oil-wet rocks in the sense that they decrease the cross-sectional area of water available in the pores for current flow. Foam appears to have competing and opposite effects caused by the presence of clay, micropores, and conducting minerals, which tend to lower the saturation exponent at low *S_w_*. Finally, the *P_c_* curve is consistently lower in foam than without foam for the same *S_w_*.

## 1. Introduction

**Figure 1:**
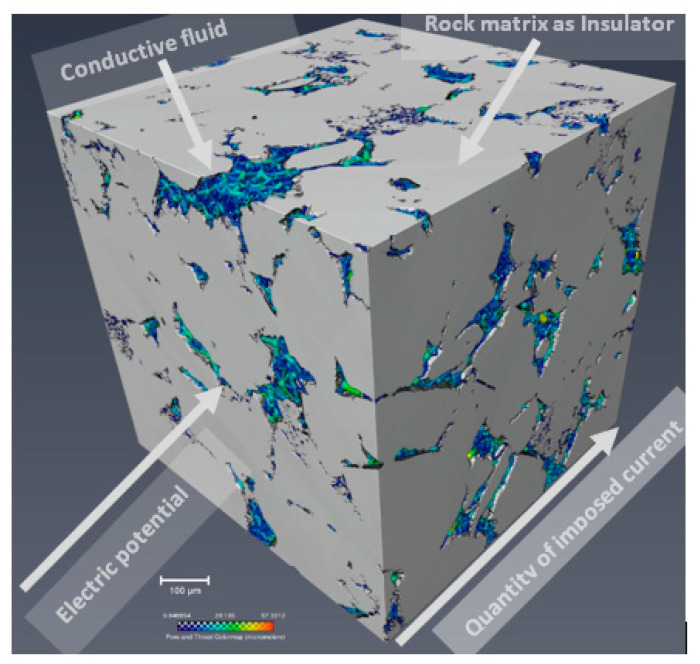
A 3D view of a rock showing the boundary conditions and components of the electrical conductivity.

Electrical resistivity index (*RI*) and capillary pressure (*P_c_*) curves are used in many reservoir-engineering and hydrology applications such as the determination of initial reservoir fluids contacts, fluid transition zones, fluid typing and distribution, rock-typing, and fluid flow. The inaccurate determination of these curves can lead to the erroneous evaluation of reservoir rocks. Ohm’s law is generally employed to determine the electrical conductivity of a porous medium as given by the relation (1):

(1) $$\sigma =-\frac{I}{\Delta V},$$

where σ is the electrical conductivity of the rock system, *I* is the quantity of current, and *∆V* is the electrical potential difference across the porous medium. The electrical conductivity can be obtained by solving the conductivity equation with the appropriate boundary conditions [1]. Figure 1 is an illustration a rock with an electric potential applied at one end, the direction and quantity of the imposed current, the conductive fluid, and the rock matrix acting as an insulator. 

It is generally assumed that the only conductive fluid in a reservoir rock is the water content, while the rock matrix, oil, and gas are not conductive but act as electric insulators in the system. Archie [2] was the first researcher to identify a relationship between electrical resistivity index and saturation, while many other researchers took after him to study these relationships for many decades. Archie’s [2] equation relating the electrical resistivity of a porous medium to its water saturation is given in Equations (2) and (3).

(2) $$S_{w}^{n}=\frac{1}{RI}$$

(3) $$S_{w}={\left(\frac{Ro}{Rt}\right)}^{1/n}$$

where *RI* is the resistivity index (a ratio of the rock resistivity at a given partial water saturation to the resistivity at 100% water saturation, $\frac{Ro}{Rt}$), $S_{w}$ is the water satution, Ro is the resistivity of the porous medium when fully saturated with the conducting fluid, and Rt is the resistivity of the porous medium when partially saturated at $S_{w}$. The slope of the straight-line plot of Equation (1) or (2) gives the saturation exponent of the rock, n of the rock sample, and the average of multiple measurements of n represents the saturation exponent of an underground reservoir of similar rock type. This value is then used to convert the well log resistivity profile, obtained along a well drilled through the underground reservoirs or aquifers, into a saturation distribution profile. Various factors affect the value of the saturation exponent such as wettability, pore geometry, saturation history, rock–fluid interactions, and others as reported in the literature [3,4,5,6,7,8,9].

It is also known that various factors affect the capillary distribution of water in a rock such as the pore and throat sizes, wettability, interfacial tension, and difference in the fluids’ densities [10]. At equilibrium, capillary pressure (*P_c_*, dyne/cm^2^) is related to the difference in the pressure of the non-wetting (e.g., gas) and wetting phases (e.g., water) according to Equations (4) and (5).

(4) $$P_{c}=\frac{2\sigma_{gw}\;Cos\;\left(\theta \right)}{r}=\left(P_{g}-P_{w}\right)=\left(\rho_{w}-\rho_{g}\right)gh$$

(5) $$h=\frac{2\sigma_{gw}\;Cos\;\left(\theta \right)}{r\left(\rho_{w}-\rho_{g}\right)g}$$

where $\sigma_{gw}$ is the interfacial tension between gas and water (dyne/cm). The contact angle, *θ*, represents the wettability of the pore surface; *r* is the pore radius (cm); and $P_{w}$ and $P_{g}$ (dyne/cm^2^) represent the water and gas pressures, respectively. Similarly, $\rho_{w}$ and $\rho_{g}$ (g/cm^3^) represent the density of water and gas, respectively, while the capillary rise of water above the free water level is represented by *h* (cm).

Accurate determination of the reservoir fluid saturation, fluid contacts, and fluid distribution and flow using electrical resistivity and capillary pressure curves depends on the accuracy of the laboratory methods and the representativeness of the laboratory measurements. When evaluating the initial fluid saturation and distribution in a reservoir rock, the saturation exponent for the reservoir system must be measured in the lab using methods that honor the reservoir state and the processes that lead to the current reservoir state. For the case of a new hydrocarbon reserve, the laboratory method involves measurements of both capillary pressure and resistivity index at different water saturations (starting from 100% water saturation) while hydrocarbon is displacing the water until irreducible water saturation. This direction of change in water saturation is often called drainage (decreasing water saturation). In the context of ground water or aquifer systems, the drainage process represents the depletion of ground water normally during dry season. On the other hand, if the electrical resistivity tool is intended to measure the water distribution in an aquifer during recharge or to determine the remaining hydrocarbon in place after an enhanced oil recovery (EOR) method such as water flooding, the measurements are obtained while water is displacing the non-wetting phase until residual non-wetting phase saturation. This direction of change in saturation is called imbibition (increasing water saturation).

**Figure 2:**
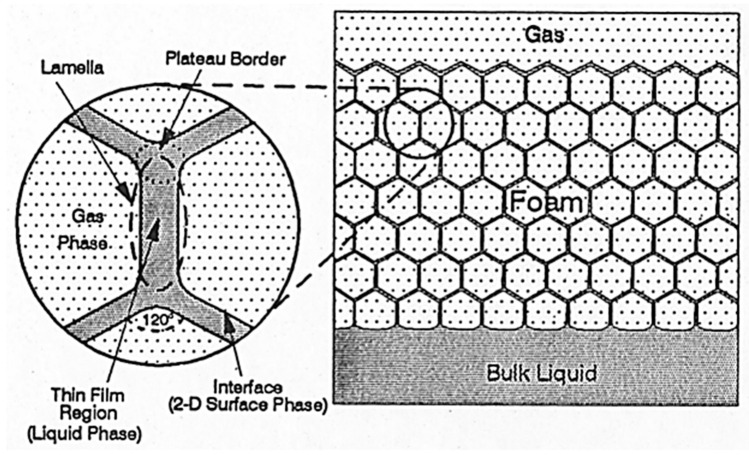
Foam system [25].

In recent years, more than half of the world’s hydrocarbon reserves have exceeded their maturity age, and their hydrocarbon production is rapidly declining. For such reservoirs, enhanced or tertiary hydrocarbon recovery methods are being considered. Such methods include injection of gas or chemicals (e.g., surfactants, polymers, CO_2_) to alter the surface tension [11,12], rock wettability, and capillary pressures. An effective alteration of the wettability and capillary pressures can significantly improve hydrocarbon recovery from tight reservoir pores that were initially un-swept during secondary recovery. In reservoirs with sections having varying ranges of permeability, the combined injection of surfactant and gas is used to generate foams that can prevent the injected gas from flowing only through the high-permeability sections. In other applications, foam is used to enhance gas injection and storage in underground reservoirs [13,14,15,16,17,18] or to have a better control of the injected fluids during the underground remediation process of contaminated soils and aquifers [19]. There are two general ways to generate foam in porous rock. One way is through the alternate injection of surfactant with gas, a process often referred to as surfactant-alternating gas [20]. Another method involves simultaneous or co-injection of the foaming solution and gas [21]. The generated foam bubbles can make a significant part of the gas phase discontinuous by thin liquid films called lamellae, separating the gas bubbles [22,23,24]. Figure 2 illustrates the components of a bulk foam.

Although Archie’s equation is based on some underlying assumptions with some associated limitations, recent researchers have extended the use of Archie’s model to monitor the propagation of foam and fluids’ distribution during foam injection in porous media [24,26,27,28]. The reliability of using Archie’s model in porous media containing foam is not yet ascertained, as Archie’s model was empirically derived for porous media having water (the wetting phase) and a non-wetting phase (oil or gas). In this study, we investigated the reliability of Archie’s model for porous media containing foamed gas and water. We also investigated the role of foam on the capillary pressure curves of these rock systems. Though, the impact of surfactant can be predicted as it is known that surfactant can reduce the surface tension between gas and water [29,30], and that foam bubbles can divert gas flow to smaller pores, but no or probably few experimental data are available to support or demonstrate how they affect capillary pressure curves. We used a porous plate method simultaneously with a two-pole electrical resistivity method to measure both capillary pressure and electrical resistivity, respectively, of some selected rock samples at different water saturations. We conducted this experiment for two fluid systems, namely, a pure gas–water system and then a foamed gas–water system, using rocks of different properties. We then compared their capillary pressure and resistivity index curves. We fitted the resistivity index data with Archie’s model for both gas–water and foamed gas–water rocks. NMR scanning was conducted on the samples at each saturation level to monitor the fluid displacement pattern in the two fluid systems.

## 2. Results and Discussions

### 2.1. Heterogeneous Composite Rock

**Figure 3:**
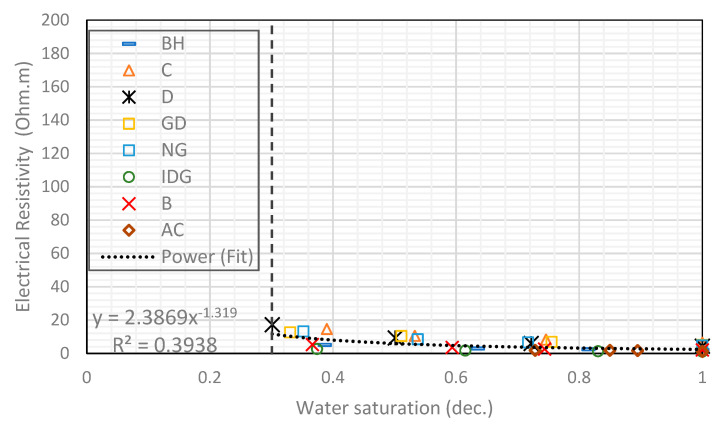
Power-law relationship between electrical resistivity and water saturation in a gas–water system.

**Figure 4:**
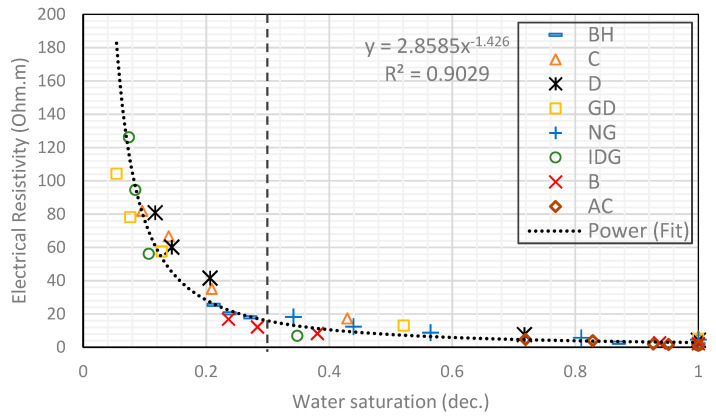
Power-law relationship between electrical resistivity and water saturation in a foamed gas–water system.

**Figure 5:**
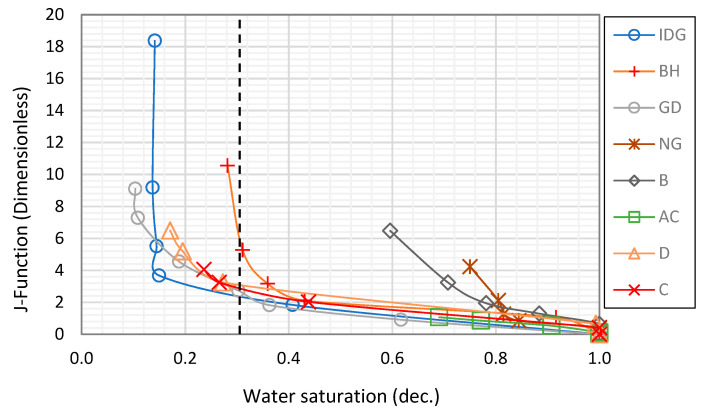
J-function curves for the composite rock.

We start by combining all eight rock samples to form a heterogeneous composite rock having a wide variety of permeability and pore geometry. Figure 3 and Figure 4 show the resistivity data versus water saturation for the composite for the pure gas–water and foamed gas–water system, respectively. The markers in each figure represent the measured data for the different rock samples that make up the composite. We then fit a power-law curve (the black dotted curve), which is the line of best fit based on the least-squares method, to the composite data, for the pure gas–water system (Figure 3) and foamed gas–water system (Figure 4). As can be observed in both figures, the irreducible water saturation (the lowest water saturation attained after displacement) for the composite rock is 0.3 (for pure gas–water system) and 0.05 (for foamed gas–water system). This is expected as foam helps to divert gas bubbles into smaller pores that could not be drained by pure gas (normally due to gravity override) and drain the water therein [25,27]. The vertical dotted lines in both figures represent the 0.3 irreducible saturation obtained with pure gas. For the foamed gas displacing water (Figure 4), the electrical resistivity of the composite rock begins to increase rapidly as the water saturation drops below this critical value (0.3). As seen in the figure, the curve kinked at this saturation value and rapidly concaved upwards. The resistivity significantly increased from about 20 to 126 Ω-m. The reason for this observation is the high resistance to electric current caused by the foam bubbles, which is higher than the electrical resistance caused by gas without foam. As pointed out earlier in the introduction (Figure 2), foam bubbles consume some of the continuous water phase in the rock to form their lamellae. The water in the lamellae provide a smaller cross-sectional area to the flow of electric current compared to the continuous water channel present in the pores. As the number of bubbles increases, the fraction of the rock water used to form foam lamellae also increases while the fraction of the less tortuous water phase decreases. This, in turn, increases the tortuous path for current flow. Below a critical water saturation, when the foam texture becomes very fine (i.e., high bubble density), the resistivity curve rapidly increased and concaved upward, as observed in Figure 4. This critical water saturation is likely dependent on the pore geometry as the foam texture tends to be finer when the foam enters tighter pores with higher capillary pressures, as shown by the J-function plots for the composite in Figure 5. The J-function attempts to average or lump the capillary pressure curves of the component rocks by accounting for the effect of porosity and permeability that would otherwise cause the curves to be different for rocks of the same rock type. As seen in the J function plots of Figure 5, the 0.3 critical water saturation (black dotted vertical line) demarcates the region of high capillary pressure (left of the vertical line) from the region of lower capillary pressures (right of the vertical line) in the composite. There appears to be two classes of J-function. The first class consists of samples BH, D, GD, NG, IDG, and AC, which can be described by a single J-function, while samples B and C appear to form another class of pore geometry. The first class of samples was generally drained below the critical water saturation (below 0.3), where the electrical resistivity begins to concave upwards. The second class of samples appeared to have a different critical saturation value (approximately 0.8).

**Figure 6:**
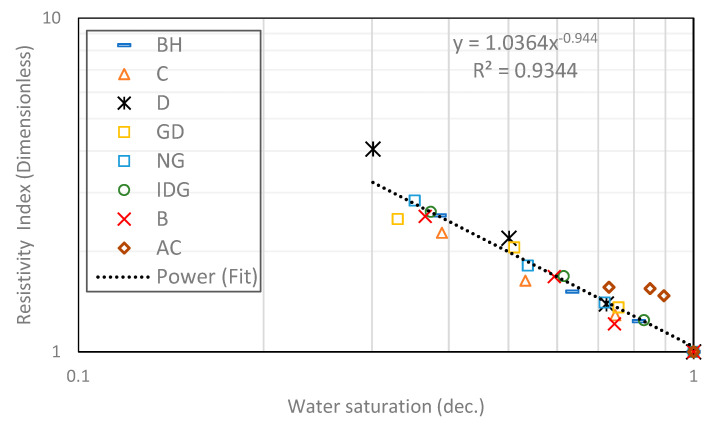
Power-law relationship between rock electrical resistivity and water saturation in a foam-water system.

**Figure 7:**
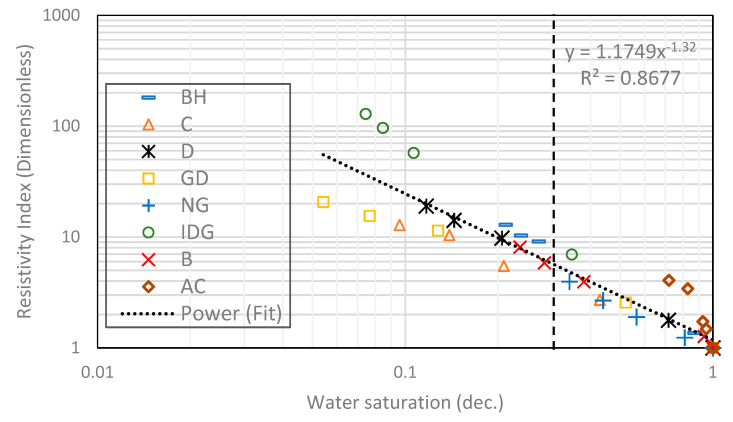
Power-law relationship between rock electrical resistivity and water saturation in a foam-water system.

Next, in Figure 6 and Figure 7, curve fitting by the least-squares method was used to fit Archie’s model (Equation (2)) to the same experimental data of Figure 3 and Figure 4, respectively, by normalizing the resistivity value of each rock in the composite with their corresponding resistivity value at 100% water saturation (R_0_). According to Archie, this plot gives a straight-line relationship with a slope representing the saturation exponent of the composite. The saturation exponent relates the impact of draining the conductive fluid on the rock electrical resistivity. For a simple, clean, and homogeneous rock, the value of *n* is constant for every drop in the water saturation, while for a complex/heterogeneous rock (like the composite in this study), *n* can change with water saturation, because of the presence of multiple pore types, as seen by some data points falling out of the straight line fit (Figure 6 and Figure 7). Later, we showed that *n* is constant at different water saturation values for each rock type in the composite. Generally, *n* is high when a rock is oil-wet and small when the rock is water-wet and/or contains sensitive minerals like clay and some conducting minerals like pyrites. The saturation exponent for the composite in this study is 0.94 for the water–gas system and 1.32 for the foamed gas–water system. In the foamed gas–water system, foam affected the saturation exponent like an oil-wet pore surface would, in the sense that it increased the value of *n*. In oil-wet rock, oil wets the pores’ surfaces and prevents water from direct contact with the pores’ surfaces. Consequently, the water phase breaks up at lower saturation into a discontinuous water channel, thereby raising the resistivity and saturation exponent of the rock. The same phenomenon plays out in the foam system as pointed out earlier. As can be seen in Figure 7, the saturation exponent appears to be very high at lower water saturation due to the high bubble density/foam texture in one of the components of the composite (sample IDG). Although IDG is a sandstone with some clay content, the effect of the clay content is dominated by the effect of the foam, thereby causing the saturation exponent to increase at lower water saturation. On the other hand, the effect of micro pores in some of the carbonate components of the composite (GD and C) seems to dominate the foam effect on the saturation exponent. Hence, *n* values are lower than those of the composite at lower water saturation. More explanation on this is given in subsequent discussions.

### 2.2. Homogenous Rocks

**Figure 8:**
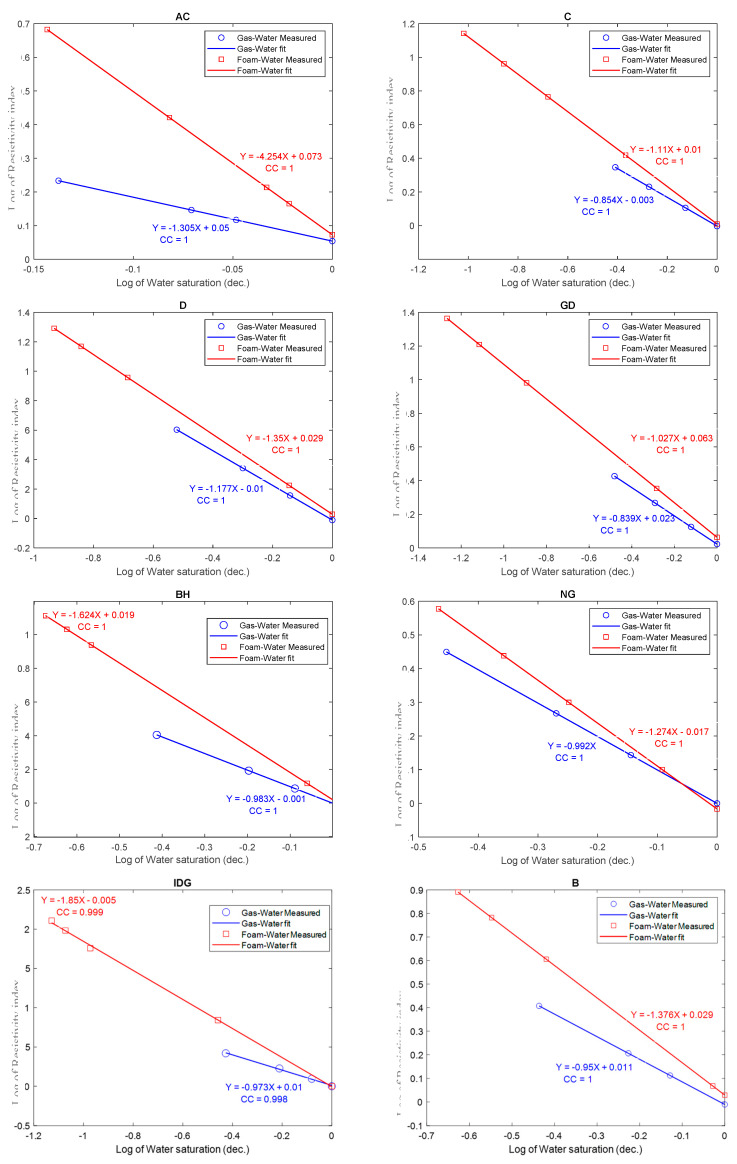
Comparison of the resistivity index curves for gas–water and foamed gas–water in different rock samples.

In this subsection, we examined the effect of foam on the individual rock samples. Figure 8 shows Archie’s model fitted to the measured resistivity index data, for both gas–water and foamed gas–water in each of the rock samples. It can be observed that the resistivity index at a given water saturation is higher in a foamed gas–water than in a gas–water system. Nonetheless, as seen in Figure 8, the relationship between the resistivity index in the foamed gas–water system can be fitted with Archie’s resistivity model (Equation (2)). The effect of foam is described by the values of the saturation exponent (slope). Hence, a higher resistivity index resulted in a higher saturation exponent in foamed gas–water than in gas–water systems. This is consistent for all the eight different samples measured. Table 1 tabulates the saturation exponents of the different rocks and the composite for both gas–water and foamed gas–water systems.

**Figure 9:**
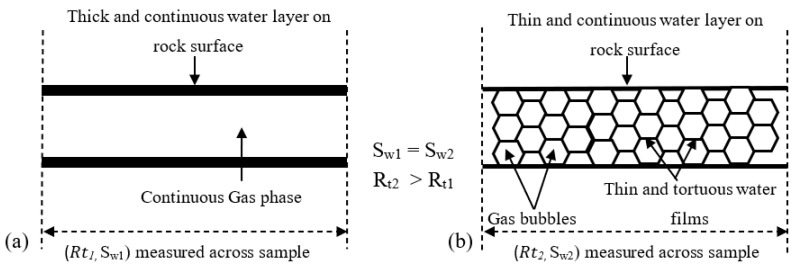
Illustration of electrical resistivity for: (**a**) Gas–water system; (**b**) foamed gas–water system.

Figure 9 is a pictorial illustration that attempts to explain why the electrical resistivity of a foam-water system is higher than that of a gas–water system. Figure 9a is a representation of a rock having a thick layer of water (the wetting phase) depicted as the two adjacent black lines. The water saturation (*S_w_*_1_) is represented by the thickness of the black lines. In between the black lines is the gas phase (the non-wetting phase). The electrical resistivity across the rock sample is denoted by *Rt*_1_. Figure 9b represents the same rock sample having a water saturation (*S_w_*_2_) whose value is the same with *S_w_*_1_. The gas phase in this case is discretized into micro bubbles. The micro bubbles have thin water films that separate them. The water films are extracted from the continuous and straight water layer (with large cross-sectional area) in the rock. Hence, the continuous water layer is thinner than that in Figure 9a. The micro bubbles also make the flow path of electrical current more tortuous and extremely thin compared to that in Figure 9a (gas–water), causing increased electrical resistivity. For this reason, the resistivity in Figure 9b (foamed gas–water) is greater than that in Figure 9a (gas–water), even when the water saturation in the rock is the same in both cases. Hence, the resistivity index is higher in the foam-water system than in the gas–water system, as presented in Figure 9.

Foam appears to affect the connectivity and electrical resistivity of a porous medium in a similar way to wettability, as studied by Liu et al. [31]. Foam like oil wetness can increase the value of the saturation exponent at lower water saturation, when the water film becomes thin, tortuous, and sometimes discontinuous.

It is important to reiterate that this study looked at the effect of foam in a general sense. However, the type of surfactant used to generate the foam in a porous medium can affect the electrical resistivity and capillary pressure by virtue of the volume and strength of the foam. Larger foam volume will cause higher resistivity. A stronger foam will also cause more gas to be trapped in the porous medium and, hence, lower irreducible water saturation and electrical resistivity. Some foam will collapse at high temperature and high salinity. When collapse occurs, the system will behave like a water–gas system.

### 2.3. Effect of Foam on Capillary Pressure Curve

**Figure 10:**
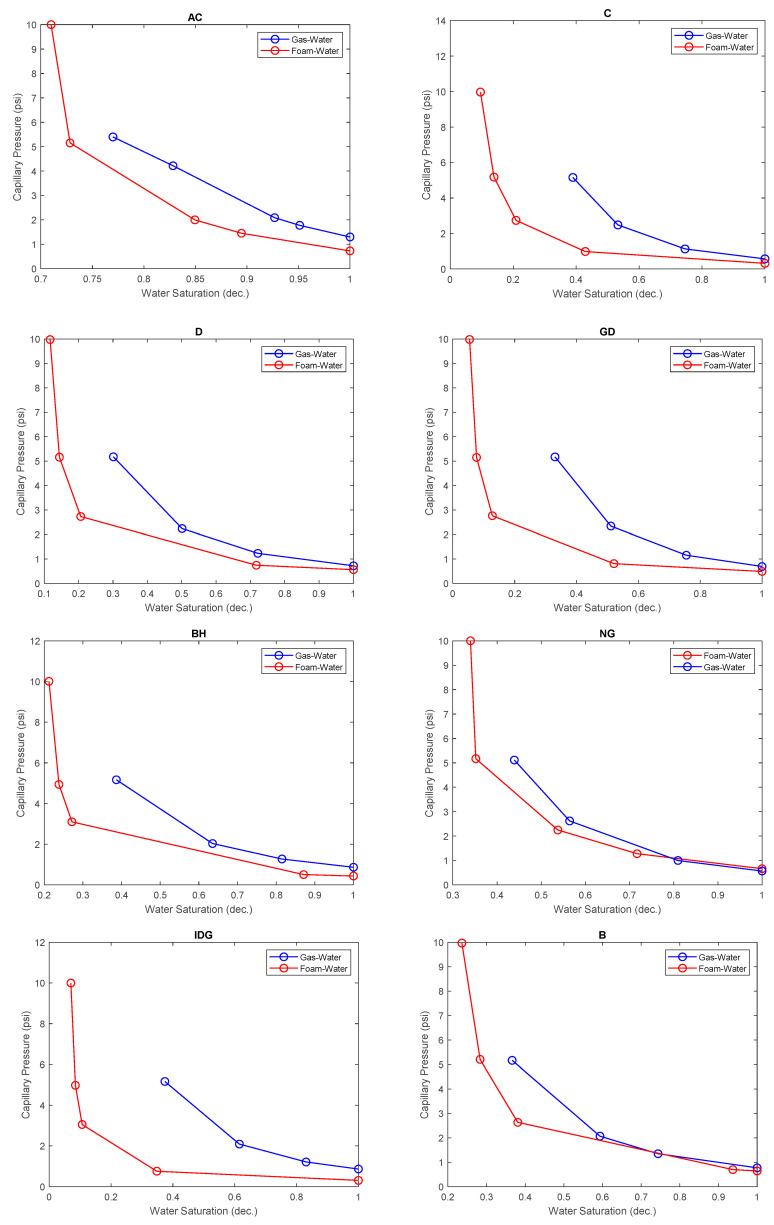
Comparison of the capillary pressure curves for foam–water and gas–water in different rock samples.

In Figure 10, the capillary pressure curve is compared between the foam displacing water and gas displacing water for each rock sample. Take note that the data points were simply connected by lines and not fitted with any model. The effect of the surfactant added to the water manifests as a corresponding reduction in the capillary pressure for every water saturation value. This is the case for all the rock types investigated, as shown in the figure. The reduction in capillary pressure consequently caused lower residual water saturation than observed in the gas-displacing-water process. This is because the surfactant foams allow smaller pore sizes to be drained by lowering the capillary pressures [32] and diverting gas flow from the big pores to the smaller pores. As a result of early gas breakthrough, the gas–water capillary pressure could not be conducted at higher capillary pressures, and, hence, lower water saturation could not be attained. As the porous media were pre-saturated with slugs of surfactant solution followed by slugs of gas injection (known as surfactant-alternating gas, as practiced in the oil and gas field), the surface tension between the pore water and the injected gas is reduced, causing a lowered capillary pressure and increased capillary number. Lowered capillary pressure and high capillary number help to mobilize and displace more water from the porous medium. The ensuing foam bubbles also help to divert subsequent gas slugs into higher-capillary-pressure pores, and more water is displaced.

### 2.4. NMR Measurements of Water Distribution

In this subsection, we used NMR relaxometry to study the distribution of water in the rock samples at three different states, namely, 100% water saturation, after gas displaced water, and after foamed gas displaced water. NMR measurements were plotted as a probability density function (PDF) of the relaxation time (*T*_2_). The PDF plot is related to the pore size distribution of the rock according to Equations (6) and (7).

(6) $$\frac{1}{T_{2}}=\rho \frac{S}{V}$$

(7) $$\frac{1}{T_{2}}=\rho \left(\frac{2}{r}\right)$$

where *T*_2_ is the relaxation time (seconds) of the hydrogen molecules in a given pore of a rock, *ρ* is the pore surface relaxivity (micrometer per second), *S* is the surface area (µm^2^) of the pore, *V* is the volume (µm^3^) of the pore, and *r* is the pore radius (µm). As such, the PDF profile can be considered a representation of the pore size distribution of a rock sample.

**Figure 11:**
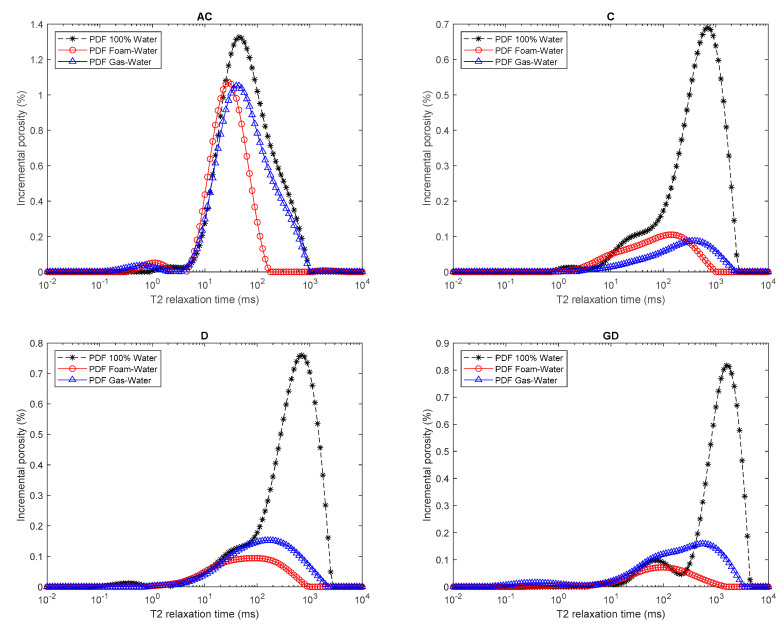

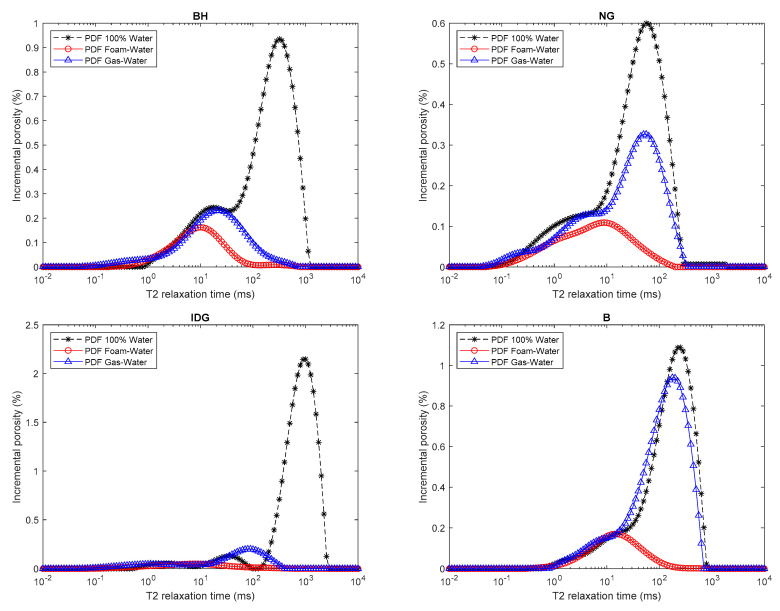
Comparison of the NMR T_2_ profile after gas and foamed gas injection in different rock samples initially saturated with water.

Figure 11 shows the combination of PDF plots of the NMR *T*_2_ relaxation times at 100% water saturation, residual water saturation after gas injection, and residual water saturation after foamed gas injection for all the rock samples. The black curves in the figures represent the water distribution at 100% water saturation, while the red curves represent the NMR measurements after the foam–gas displaced water, and the blue curves represent the NMR measurements after the gas displaced water. As indicated by the PDF curves, foamed gas drained more pores than pure gas. Smaller pores that could not be drained by pure gas (blue curves) were drained after foamed gas injection (red curves). Low *T*_2_ values in the figures represent small pores while large *T*_2_ values represent big pores according to Equation (7).

## 3. Materials and Methods

### 3.1. Sample Preparation

Eight outcrop rock samples with different petrophysical rock properties were used for this study. The rock samples were cut into cylindrical shapes and labeled BH, C, D, GD, NG, IDG, B, and AC, which cover sandstones, limestones, and dolostones. All the samples were cleaned with methanol using a solvent reflux method in order to remove all soluble salts from their pores. The samples were then dried in a vacuum oven for about 48 h at a constant temperature of 60 °C. After drying, the samples were saturated with a synthetic brine, in the first part of the experiments, and with a mixture of brine and surfactant in the second part, using a vacuum saturation method in a steel vessel. A constant pressure of 1700 psi was further applied to force more brine into the rocks to ensure 100% rock saturation. The synthetic brine was prepared by dissolving 5% wt. potassium chloride (KCl) in de-ionized water. For the second part of the experiment, the saturating brine was mixed with 0.025% wt. of a surface-active agent, a non-ionic FS-31 surfactant obtained from DuPont, Jebel Ali, Dubai, United Arab Emirates. The surfactant was used to generate foam in the rock samples. Although a non-ionic ethoxylated fluorocarbon surfactant Capstone- FS-31 was used in this study, the findings from this study generally apply to all foam in porous media regardless of the type of surfactant used to generate them. Table 2 shows the dimensions, porosities, and permeabilities of the rock samples used for this study.

### 3.2. Experimental Apparatus

**Figure 12:**
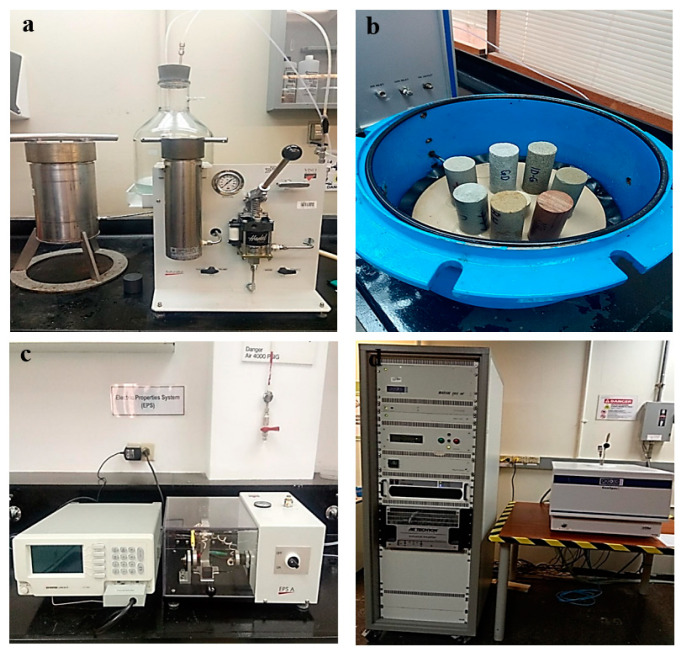
(**a**) Vacuum saturator; (**b**) porous plate capillary pressure cell; (**c**) electrical resistance meter; (**d**) NMR Geospec 2.1 rock analyzer.

The experiments were conducted using the apparatus shown in Figure 3. The apparatus include a vacuum saturator (Figure 12a) for saturating the rock samples, a capillary pressure cell (Figure 12b) for de-saturating the samples and measuring the capillary pressure curves, an electrical resistance meter (Figure 12c) for measuring the electrical resistivity of the samples at full and partial water saturations, and an NMR Geospec 2.1 rock analyzer, manufactured by Oxford Instruments, United Kingdom (Figure 12d) for measuring the fluid desaturating path in the different pore sizes based on NMR *T*_2_ relaxometry. All experiments were conducted at atmospheric temperature.

### 3.3. Experimental Procedures

For the first part of the experiment, the cleaned rocks were saturated with synthetic brine containing 5% wt. KCl. The resistivity of each sample at 100% saturation was first measured before placing the cores samples in the capillary pressure cell. All eight samples were placed in the capillary pressure cell as a single batch, as shown in Figure 3b. A high-purity (99.9%) nitrogen gas was then used to simultaneously de-saturate the samples at capillary pressure (*P_c_*) values of 1, 3, 5, and 10 psi. The fluids expelled from the samples were collected in a tight sealed flask sitting on a high-resolution weight balance (with an accuracy of 0.001 g). As fluid was expelled from the capillary pressure cell, the weight of the fluid was measured by the weight balance. The capillary pressure was raised to the next pressure step after equilibrium was achieved in the cell. Equilibrium was adjudged to be attained when the fluid production and weight of the produced fluid remained constant for two consecutive days. At equilibrium, the samples were taken out and their weights measured to determine the samples’ water saturation (*S_w_*) at the imposed capillary pressure value. The electrical resistivity (Rt) across each sample was also measured to determine the resistivity index at this saturation value. The electrical resistance meter has a two-pole electrodes configuration. The capillary pressure was then increased to a higher value and the procedures were repeated for the different capillary pressure values in order to generate complete capillary pressure (Pc–*S_w_*) and electrical resistivity index (RI–*S_w_*) curves for all the samples. The experiment was completed after six weeks. The entire procedure was then repeated for the second part of the experiment, where the saturating brine contained 0.025% wt. surfactant that served as the foaming agent. The surfactant concentration used is the critical micelle concentration (CMC), i.e., the concentration above which further surfactant concentration will not yield further change in the surface tension. It should be noted that the choice of surfactant type does not affect this study, as the objective is to study the general effect of the foam generated on the electrical resistivity and capillary pressure of the rocks containing them. This aspect of the experiment also lasted about eight weeks. In this aspect, the dissolved surfactant in the water phase reduced the surface tension between the injected gas and brine during drainage. Foam bubbles are also formed in the process as one of the mechanisms of foam generation is through gas displacement of surfactant solution in a porous medium [15,20].

The low-magnetic-field (2 MHz) NMR system instrument) was used to measure the *T*_2_ relaxation of the samples at 100% water saturation and after every capillary pressure value using the (Carr-Purcell-Meiboom-Gill) CPMG pulse sequence. The NMR scanning parameters are: Tau value of 0.1 ms; signal-to-noise ratio of 100–200, and a recycle delay of 11,250 ms.

## 4. Conclusions

This paper investigated the effects of foam bubbles on the electrical properties and capillary pressures of rocks. The following conclusions are drawn from the results of this study.

1. Foam bubbles use some of the rock pore water to form thin water films called lamellae. As a result, the volume of the straight and continuous (with large surface area) water phase is reduced while the water phase in the lamellae makes the electric current path more tortuous. Hence, at the same water saturation and for the same rock, the electrical resistivity is higher for a foam–water than for a gas–water system.
2. The electrical tortuosity (caused by foam bubbles) increases as the total water saturation of the rock decreases. Below a critical water saturation, the tortuosity increases exponentially. Hence, the relationship between electrical resistivity and water saturation exhibits a power-law behavior in a foam-water system.
3. Archie’s model can fit the experimental data from foam. However, the saturation exponent for a foamed gas–water system is consistently higher than that of a pure gas–water system.
4. Generally, foam affects the saturation exponent like an oil-wet pore surface would, in the sense that it increases the value of *n* at lower water saturation. There is a competing and opposite effect on the saturation exponent by the presence of foam and the presence of clay, micropores, and conductive minerals in a porous medium. Foam tends to increase the saturation exponent while clay, micropores, and conductive minerals tend to decrease it.
5. The presence of a surface-active agent such as surfactant reduces the surface tension between gas and water. This causes the capillary pressure curve at a given water saturation to be lower in a foam–gas system than in a gas–water system. The presence of foam also allows smaller pores to be drained, resulting in lower irreducible water saturation.

## Figures and Tables

**Table 1:** Saturation exponents for gas–water and foamed gas–water in different rock samples.

Sample	Lithology	*k*	*φ*	Saturation Exponent ‘*n*’	
		(mD)	(%)	Gas–Water	Foam–Water
AC	Austin Chalk	26	26.89	1.305	4.254
C	Silurian Dolomites	136.61	12.81	0.854	1.110
D	Dolomite	327.83	15.29	1.177	1.351
GD	Guelph Dolomite	637.26	20.85	0.839	1.027
BH	Brill Hill Sand	1100	18.92	0.983	1.624
NG	Nugget sand	111.32	12.3	0.992	1.274
IDG	Idaho Grey sand	5000	29.2	0.973	1.850
B	Berea sand	270	20	0.960	1.376
Composite	Heterogenous	-	-	0.944	1.32

**Table 2:** Dimensions and petrophysical properties of core samples.

Sample	Lithology	*L* (cm)	*D* (cm)	*k* (mD)	*φ* (%)
AC	Austin Chalk	3.559	3.787	26	26.89
C	Silurian Dolomites	8.643	3.748	136.61	12.81
D	Dolomite	9.337	3.744	327.83	15.29
GD	Guelph Dolomite	9.771	3.801	637.26	20.85
BH	Briarhill Sand	9.907	3.760	1100	18.92
NG	Nugget sand	9.776	3.791	111.32	12.3
IDG	Idaho Grey sand	10.09	3.780	5000	29.2
B	Berea sand	9.267	3.795	270	20
